# Reef calcifiers are adapted to episodic heat stress but vulnerable to sustained warming

**DOI:** 10.1371/journal.pone.0179753

**Published:** 2017-07-06

**Authors:** Marleen Stuhr, Claire E. Reymond, Vera Rieder, Pamela Hallock, Jörg Rahnenführer, Hildegard Westphal, Michal Kucera

**Affiliations:** 1 Department of Biogeochemistry and Geology, Leibniz Centre for Tropical Marine Research (ZMT), Bremen, Germany; 2 Department of Statistics, TU Dortmund University, Dortmund, Germany; 3 College of Marine Science, University of South Florida, St. Petersburg, Florida, United States of America; 4 Department of Geosciences, University of Bremen, Bremen, Germany; 5 MARUM, Center for Marine Environmental Sciences, University of Bremen, Bremen, Germany; Universita degli Studi di Urbino Carlo Bo, ITALY

## Abstract

Shallow marine ecosystems naturally experience fluctuating physicochemical conditions across spatial and temporal scales. Widespread coral-bleaching events, induced by prolonged heat stress, highlight the importance of how the duration and frequency of thermal stress influence the adaptive physiology of photosymbiotic calcifiers. Large benthic foraminifera harboring algal endosymbionts are major tropical carbonate producers and bioindicators of ecosystem health. Like corals, they are sensitive to thermal stress and bleach at temperatures temporarily occurring in their natural habitat and projected to happen more frequently. However, their thermal tolerance has been studied so far only by chronic exposure, so how they respond under more realistic episodic heat-event scenarios remains unknown. Here, we determined the physiological responses of *Amphistegina gibbosa*, an abundant western Atlantic foraminifera, to four different treatments––control, single, episodic, and chronic exposure to the same thermal stress (32°C)––in controlled laboratory cultures. Exposure to chronic thermal stress reduced motility and growth, while antioxidant capacity was elevated, and photosymbiont variables (coloration, oxygen-production rates, chlorophyll *a* concentration) indicated extensive bleaching. In contrast, single- and episodic-stress treatments were associated with higher motility and growth, while photosymbiont variables remained stable. The effects of single and episodic heat events were similar, except for the presumable occurrence of reproduction, which seemed to be suppressed by both episodic and chronic stress. The otherwise different responses between treatments with thermal fluctuations and chronic stress indicate adaptation to thermal peaks, but not to chronic exposure expected to ensue when baseline temperatures are elevated by climate change. This firstly implies that marine habitats with a history of fluctuating thermal stress potentially support resilient physiological mechanisms among photosymbiotic organisms. Secondly, there seem to be temporal constraints related to heat events among coral reef environments and reinforces the importance of temporal fluctuations in stress exposure in global-change studies and projections.

## Introduction

The health and the geographical distribution of coral reefs are rapidly declining with ever increasing local and global pressures [1]. Among the most prominent causes for this decline is long-term ocean warming, often manifested as transient heat events, which induce the loss of photosynthetic microalgae and/or photopigments from reef organisms, known as bleaching [2]. The bleaching phenomenon was first observed among corals [2, 3] and has since been documented among other photo-symbiotic tropical organisms including large benthic foraminifera (LBF) [4]. In recent years shallow-water tropical reef regions (e.g., the Great Barrier Reef) have undergone massive bleaching events [5], which are expected to become regular occurrences in the coming decade [6]. The ongoing decline of coral populations and degradation of coral reefs has kindled interest in the thermal tolerance, adaptive value and stability of algal-invertebrate symbioses in these environments under higher temperature regimes [7, 8].

The LBF *Amphistegina* spp. is a circumglobal, warm-water, calcifying eukaryote inhabiting oligotrophic coral-reef and shallow-shelf environments and hosting diatom photosymbionts [9]. Facilitated by their algal symbionts, LBF are vital constituents of coral-reef ecosystems [10] and important marine calcifiers, responsible for the global production of approximately 0.1 Gt/year of carbonate sediments [11]. Due to their physiological sensitivity, LBF are commonly used as bioindicators for past and present environmental conditions such as water quality and coral reef health [12, 13]. The LBF are exceptionally useful models for studying the effects of global change on marine photosymbiotic calcifiers, primarily due to their abundance, fast growth, and easy handling in culture. Previous studies have shown that extreme and chronic thermal stresses have direct detrimental effects on calcification and overall host and photosymbiont (i.e., holobiont) fitness [14–17]. These studies have characterized either the immediate response to elevated temperatures or the effects of chronic exposure. Yet, how LBF react to episodic stress events, followed by intervals of thermal respite, is currently unknown. This is a vital aspect of adaptive physiology, because episodic stress followed by a phase of recovery, represents a realistic scenario for predicting the consequences of present and future global warming [18].

Thermal stress appears to affect LBF primarily by impairing the function of the photosynthetic apparatus of the algal symbionts [15, 17, 19]. Such impairment can include reduced expression of the rate-limiting carbon-fixation enzyme RuBisCO (ribulose 1-5-biphosphate carboxylase/-oxygenase) [20], reduced photopigment concentrations and photosynthetic performance [14–17, 21, 22] and reduced oxygen-production rates [16]. Collectively, thermal stress can cause reduced growth, calcification, survivorship and fecundity [14–16, 22–24], as well as host inactivity [17]. The exact kinds of molecular damage and cellular stress-related mechanisms that mediate these effects remain unknown. Similarly, the processes of recovery of LBF after stress exposure have not been previously reported. Recovery potential, however, is important in the context of episodic stress exposure, as such potential may facilitate survival despite peak temperatures reaching the bleaching threshold, and could even increase thermal tolerance [7, 8]. Recovery responses could explain how LBF thrive in habitats where local temperatures can exceed temperatures that induce mortality when persistent over several days [13].

The local effects of global warming include fluctuating physicochemical conditions across spatial and temporal scales [18, 25]. In response to dynamic atmospheric and hydrographic processes, including cloud formation, wind-driven advection, diurnal heating and cooling, tides and internal waves, many abiotic parameters (e.g., intensity of solar irradiance, pH, temperature, and nutrient availability) can be altered on scales from hours to weeks [7, 26–28]. Such fluctuations can be experienced from the surface of the ocean to mesophotic depths within coral-reef habitats [29, 30]. For example, the Florida Keys already experience high levels of thermal stress on a near-annual basis [6]. Common daily subsurface temperature fluctuations here are on the order of 2 to 5°C [31], but peak within-day ranges during summer can reach as much as 7 to 9°C at 20 to 30 meters depth, respectively [29]. Environmental heterogeneities influence the sensitivity of organisms to changing ocean conditions [18] and should be considered when assessing their thresholds and tolerances. For instance, when temperature fluctuations are incorporated into model projections of global warming scenarios, the effects on species performance are stronger [25], highlighting the necessity to understand resilience to episodic stress events.

In this study, we investigated how the effects of episodic exposure to thermal stress, followed by recovery phases of thermal respite differ from the effects of chronic exposure to heat stress in LBF. Along the lines of earlier studies conducted on corals [8, 26, 27, 32], our hypothesis emphasizes the role of thermal variations on the physiological performance of LBF. Specifically, we carried out a laboratory-based culturing experiment, exposing the common western Atlantic LBF species *Amphistegina gibbosa* to one of four treatments, (a) control, (b) a single thermal-stress event, (c) episodic thermal-stress events or (d) chronic thermal stress. Our goals were to determine (i) whether single or episodic event exposure to thermal stress causes similar physiological response as chronic exposure, (ii) if the initial physiological response recovers after the stress is released and (iii) if acclimatization occurs to repeated short-term stress events.

## Materials and methods

### Sample collection and preparation

**Fig 1:**
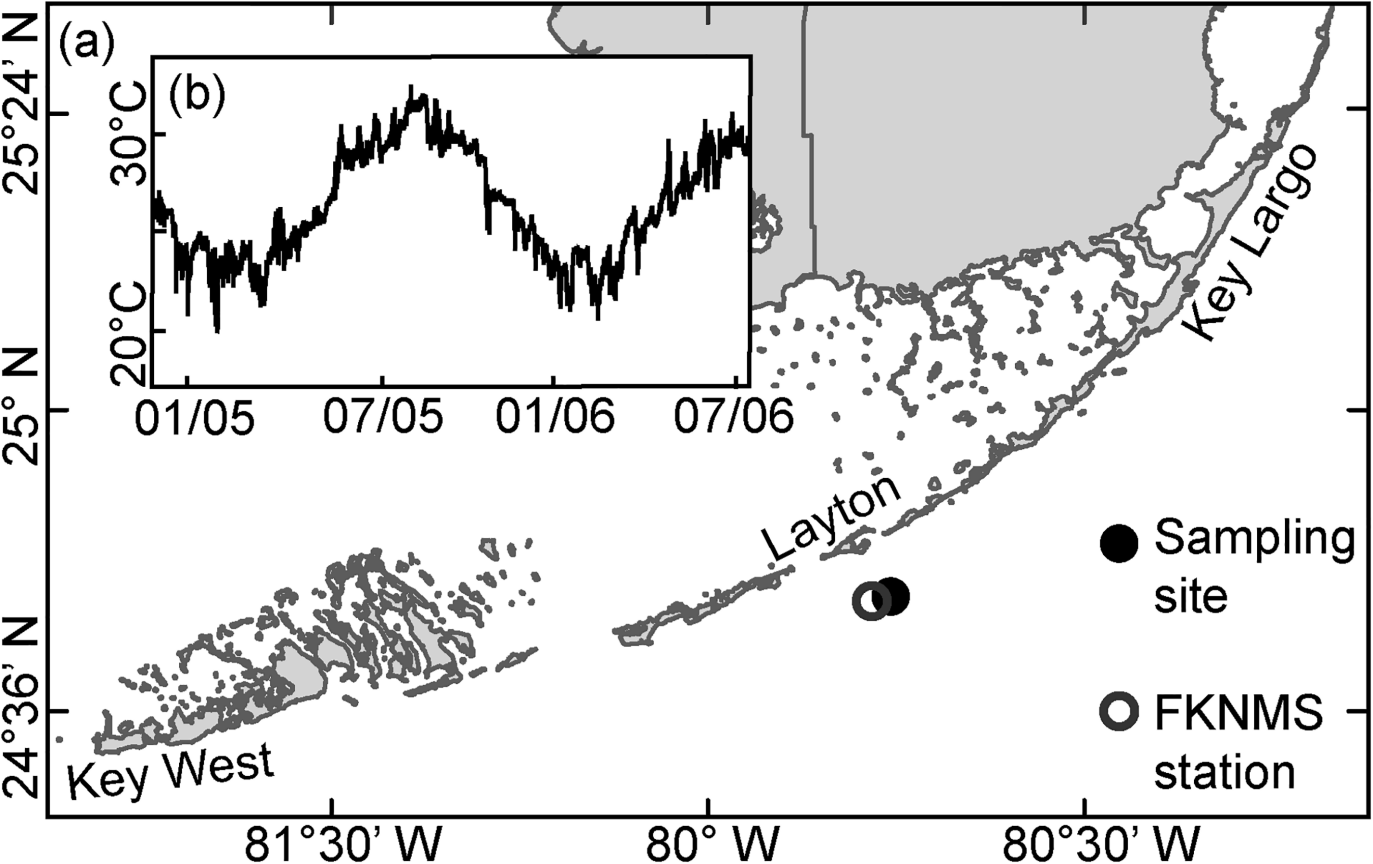
Map of the sampling location and local bottom water temperature measurements. (a) Map of the Florida Keys, USA, indicating the sampling site at Tennessee Reef in 18 m depth and the Florida Keys National Marine Sanctuary (FNKMS) station in 5 m depth where the bottom water temperature was continuously measured from 2004 to 2006; (b) Bottom water temperature at FKNMS station Tennessee Reef shows temperature fluctuations from 20°C in winter to above 30°C in summer [34].

For this study *A*. *gibbosa* were collected from 18 m depth at Tennessee Reef in the Florida Keys (Fig 1A), North Atlantic (24°45'8.33"N, 80°45'26.33"W), in June 2015. The site was previously described [6, 33] and sampling followed established protocols [9]. Sea-surface temperature (SST) in Florida ranges from winter minima of ~21°C to summer maxima of ~31.5°C with mean values of ~26.5°C [29]. *In situ* temperature measurements are slightly cooler than satellite-derived SST estimates [28] and mean temperature decreases with depth [31]. The closest long-term monitoring station to our sampling site is at the Tennessee Reef Station managed by the Florida Keys National Marine Sanctuary (FKNMS) at 5 m depth (Fig 1B) [34]. The temperature trends logged here reflect similar *in situ* bottom water temperatures, however unfortunately no long-term measurements are known for deeper areas but are assumed to be slightly cooler at 18 m depth.

After sample preparation at the Keys Marine Laboratory (KML, Layton, USA) and sorting species in the laboratory of the University of South Florida (USF, St. Petersburg, USA), specimens were shipped (inside insulated containers for <24 h) to aquaria in Bremen, Germany. During preparation and transportation, temperature loggers (Hobo, Onset, USA) recorded an average temperature of 24.99 ± 0.27°C. All specimens were acclimated to 25.5 ± 0.5°C under 5–10 μmol photons m^-2^ s^-1^ on a 12-h light/dark cycle for three weeks prior to the initiation of the experiment, approximating mean baseline temperature appraised for their natural habitat at 18 m depth and ‘stock-culture’ conditions [19]. Synthetic seawater (Tropic Marine Sea Salt, Germany) was used and maintained at a salinity of 35.5, all specimens were fed 15 μl of autoclaved microalgae [21] every nine days.

### Mimicking thermal stress

Our experiment utilized a setup modified from [21] that incorporated 12 independent aquaria (working volume 18 l). Previous experiments with *Amphistegina* spp. in culture have shown that large water volume and water movement are beneficial for extended maintenance, most likely as such setup mimics natural conditions better than small-volume stationary culture dishes [9]. For each experimental scenario, three randomly allocated replicate aquaria were used containing ~80 individuals of *A*. *gibbosa*. Each aquarium was equipped with a temperature sensor, titanium heating rod, and an aquarium pump (Nanoprop 5000, Aqua Medic, Germany). Temperature was controlled automatically with an AT-Control system (Aqua Medic, Germany) and logged with a HOBO data logger (Model UA-002-64, Pendant, Onset, USA) per treatment. Salinity, pH, and temperature were measured every second day in all aquaria, using a multimeter (WTW, Germany). Experimental light levels were set to ~5–10 μmol photons m^-2^ s^-1^ on a 12-h light/dark cycle, supplied by tri-chromatic daylight fluorescent glow tubes (T5 Reef White 10K 54W, Aqua Medic, Germany) and regulated by a light controller unit (Aqua Medic, Germany). Light levels inside the aquaria were recorded at the beginning and the end of the experiment using a light quantum meter (LI-COR LI-250A) with a submersible micro-quantum sensor (Waltz, Germany). Within each aquarium, several subsets of foraminifers were kept in glass vials, covered with a 400 μm nylon mesh to allow the water to circulate into the vial but keep the specimens in their vials throughout the experiment.

**Fig 2:**
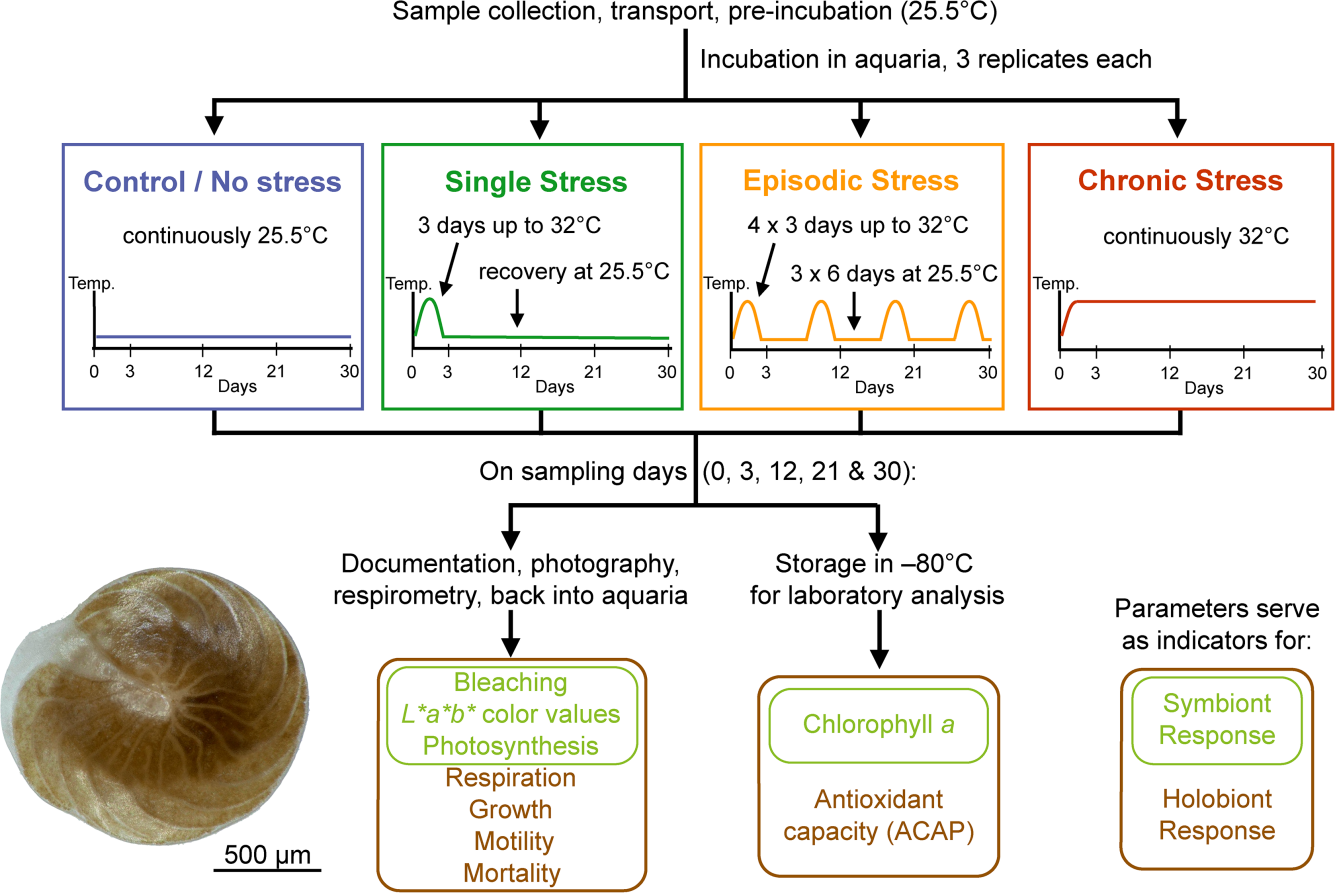
Flowchart illustrating the experimental setup including the four treatments and measured variables. Each experimental variable is shown as either indicating the symbiont or the holobiont response. The photo in the lower left corner shows a photo of an adult *Amphistegina gibbosa* specimen, taken with a digital microscope (VHX-5000, Keyence, Germany).

With the expectation that the changes in photosymbiont- and holobiont-specific response variables can provide insights into the interactions between the symbiotic partners over time and after repeated exposure, we monitored them regularly throughout the experiment (Fig 2). The photosymbiont parameters assessed included photopigment concentration, bleaching frequency, photosynthetic rates and changes in coloration. The impact on overall fitness of the LBF, the onset of oxidative stress on the holobiont level and disruption in calcification, were assessed by motility, mortality, antioxidant capacity against peroxyl radicals (ACAP), respiration and growth rates. A fixed subset of five *A*. *gibbosa* specimens from each aquarium was used to record these variables the day before the experiment and after each episodic temperature stress event, which occurred (i.e., on days 0, 3, 12, 21 and 30.) Over three days the temperature peaks were simulated by slowly raising the temperature by ~0.25°C per hour for the first 24 h, for the second day keeping it at 32°C and on the third day, slowly letting it drop back to control temperature of 25.5°C. In the chronic stress treatment, the temperature was raised similarly in the first day of the experiment, but kept at 32 ± 0.5°C until the end of the experiment. Specimens that appeared dead or experienced mechanical damage during the experiment were excluded from further data analysis, but remained within the experiment. For Chl *a* and ACAP analyses, separate subsamples were taken from all aquaria on each sampling day and immediately frozen at –80°C until further processing.

### Motility and growth

Motility is an indicator for the foraminifers’ activity and fitness [17]. By means of their reticulopodial network, the specimens were able to climb the walls of the glass vial and attach to the mesh covering the vial. On each sampling day, the location of all specimens within the glass vial was documented and rated by the distance they moved since the previous sampling: on the bottom ‘1 = Low’, on the wall ‘2 = Medium’ or on the mesh ‘3 = High’. The average motility in each vial was estimated by the following equation:

$$\text{Motility index}=\frac{\left({1∙\text{N}}_{\text{bottom}}+2∙{\text{N}}_{\text{wall}}+{3∙\text{N}}_{\text{mesh}}\right)}{{\text{N}}_{\text{total}}}\;N=\mathrm{number}\;\mathrm{of}\;\mathrm{specimens}.$$

To determine the growth rates and coloration, high-resolution photographs were taken on a standardized color background (RAL 4007-P) using fixed settings and stable light conditions with a Zeiss Discovery V8 SteREO Microscope connected to a Canon EOS 600D camera. The surface area of each specimen was measured in photographs via the software Fiji v2.0.0 [35]. A precision of 1% was predetermined for this method by repeated measurements of five specimens 20 times. Growth was estimated as an increase in cross-sectional surface area (mm^-2^) of all surviving foraminifera in comparison to the previous measurement and calculated as growth per day (% d^-1^) [36].

### Mortality and bleaching

As described by [37], empty shells reflect mortality, which is either due to stress-induced death, natural causes (old age), or reproduction. Our sampling strategy and the contorted form of the vials inhibited detection of any juveniles, which resulted from sexual or asexual reproduction. The outermost (newest) chamber normally lacks symbionts (see image in Fig 2). When any older chambers were pale or showed white spots, these specimens were recorded as mottled or partly bleached [19]. The means and SE of the proportions (n = 3) of mortality as well as partial bleaching (mottling) of the surviving specimens out of the 5 initially pooled individuals were calculated.

### Holobiont color

The holobiont color was determined using the CIE L*a*b* color space values of each foraminifer after Hosono et al. [38]. In each image, holobiont color and background color were transformed into CIE L*a*b color space by using the color space converter in Fiji [39] and determined with the same software. Artifacts of light reflecting on the shiny foraminifera shells were excluded. Mean color values were corrected by the color determined for the standardized color pallet in the image (L* = 30.24, a* = 12.25, b* = -5.47) [38]. The resulting color values represent the three coordinates within the CIE L*a*b* color space: L* indicates whiteness (brightness) of the color (0 = black, 100 = white), a* indicates the position between green (negative values) and magenta (positive values), and b* indicates the position between blue (negative values) and yellow (positive values).

### Respiration and net photosynthesis

Respiration was determined by measuring the oxygen concentration in custom-made (~1 ml) respirometry chambers with 400–600 μm diameter oxygen micro-sensors (OX-MR, Unisense, Denmark). During the measurements, each chamber housed the fixed subset of five *A*. *gibbosa* and one chamber with only seawater served as a control for background respiration. The same specimens were used for each repeated measurement throughout the experiment. Micro-sensors were introduced into the airtight glass vials containing a magnetic stirrer and the foraminifers, separated by a mesh net. To keep the water temperature stable within the vials, they were submerged in a temperature-controlled water bath set to 25.5°C except for the chronic treatments specimens, for which the temperature was set to 32°C. After a dark acclimation phase of 45 min, respiration was determined during a 30-min dark phase, followed by a 30-min light phase for net photosynthesis measurements, as adapted from previous studies [17, 40, 41]. During light incubations, light intensities replicated the conditions used during the experiment (5–10 μmol photons m^-2^ s^-1^). Respiration (oxygen consumption) and net photosynthesis (oxygen production) rates were normalized to total surface area of the living foraminiferal specimens predetermined from photographs (as described in the previous section). The daily rates were extrapolated according to a 12-h day/night cycle and gross photosynthesis rates of each replicate were calculated by adding respiration to net photosynthesis rates.

### Chlorophyll *a* concentration

To determine the photosymbiont biomass, the concentration of Chl *a* was measured, adapted from Schmidt et al. [17] by changing the protocol from using the foraminifers’ wet weight to measuring the dry weight of the crushed specimens after extracting the pigment, reducing the potential risk of overestimating weight due to additional water. The remaining foraminiferal pellets were dried for at least 24 h at 40°C within Eppendorf vials and weighed to 0.001 mg accuracy. The resulting Chl *a* concentrations were normalized by the pellet dry weights.

### Antioxidant capacity against peroxyl radicals (ACAP)

From each aquarium, a subsample of 10 specimens was collected on each sampling day and immediately frozen (–80°C) for ACAP analysis. To evaluate the biological resistance of the LBF to peroxyl radicals, ACAP assays were performed to predict the oxyradical-mediated effect on the physiological condition of organisms. Analyses utilized a fluorescence technique [42] and adapted for foraminiferal samples [23]. This method determines the antioxidant capacity indirectly by measuring the reactive oxygen species (ROS) concentration in each sample. Thus, a high capacity to neutralize peroxyl radicals results in low ROS concentrations, indicating a high ACAP. The antioxidant capacity was calculated according to Amado et al. [42] and is expressed as the inverse of the relative area [23].

### Data analysis

A principal component analysis (PCA based on correlation matrix) of the variables bleaching, color values L*, a*, b*, Chl *a*, ACAP, growth, motility, and frequency of empty shells on days 12, 21 and 30 was conducted using Past v3.11 [43]. All further statistical analyses were performed and figures were produced with the statistical programming software R [44], Version 3.2.4.

Repeated-measures analysis of variance (ANOVA) for related (dependent) groups of aquaria was applied for growth, motility, net photosynthesis, respiration, Chl *a*, and ACAP, to test for differences between treatments using the R function aov to fit an analysis of variance model [45]. In this model, aquarium as the blocking random factor was nested within treatment. Time (sampling day) and treatment were within and between fixed factors, respectively. With respect to the use of ANOVA, although the data distribution appeared to be normally distributed, the normality assumption could not be formally tested due to the small sample size in each aquarium. The small sample size also precluded the use of non-parametric methods.

For the binary outcomes of bleaching (mottling) and mortality, logistic regression with a random effect for aquaria was used by employing glmer in the R package lme4 [46]. By means of a likelihood ratio test, significant differences among treatments, times and the interaction between treatment and time can be tested, but due to numerical problems (quasi-complete separation), only descriptive analyses were conducted for bleaching and mortality.

Missing values due to high mortality in some replicates caused an unbalanced design. When there were a few missing values, they were imputed using the R package longitudinalData using the function imputation with method linearInterpol.locf [47] to conduct an ANOVA. Intermediate missing values were linearly interpolated whereas last observation carried forward (locf) and next observation carried backward (nocb) imputations were used for monotonic missing values (at the start and the end of the trajectories). In the case of oxygen consumption (respiration) and oxygen production (net photosynthesis), all values are missing for episodic stress at day 30 due to instrument failure. To avoid the loss of one level each in treatment and time, two analyses were performed on the datasets, the first without episodic stress and the second without day 30. To further identify individual differences between treatments within sampling times, significant ANOVAs were followed by Tukey's HSD test procedure using the R package lsmeans [48] thereby controlling for the multiple testing problem. All experimental data are available through the PANGAEA database.

## Results

**Fig 3:**
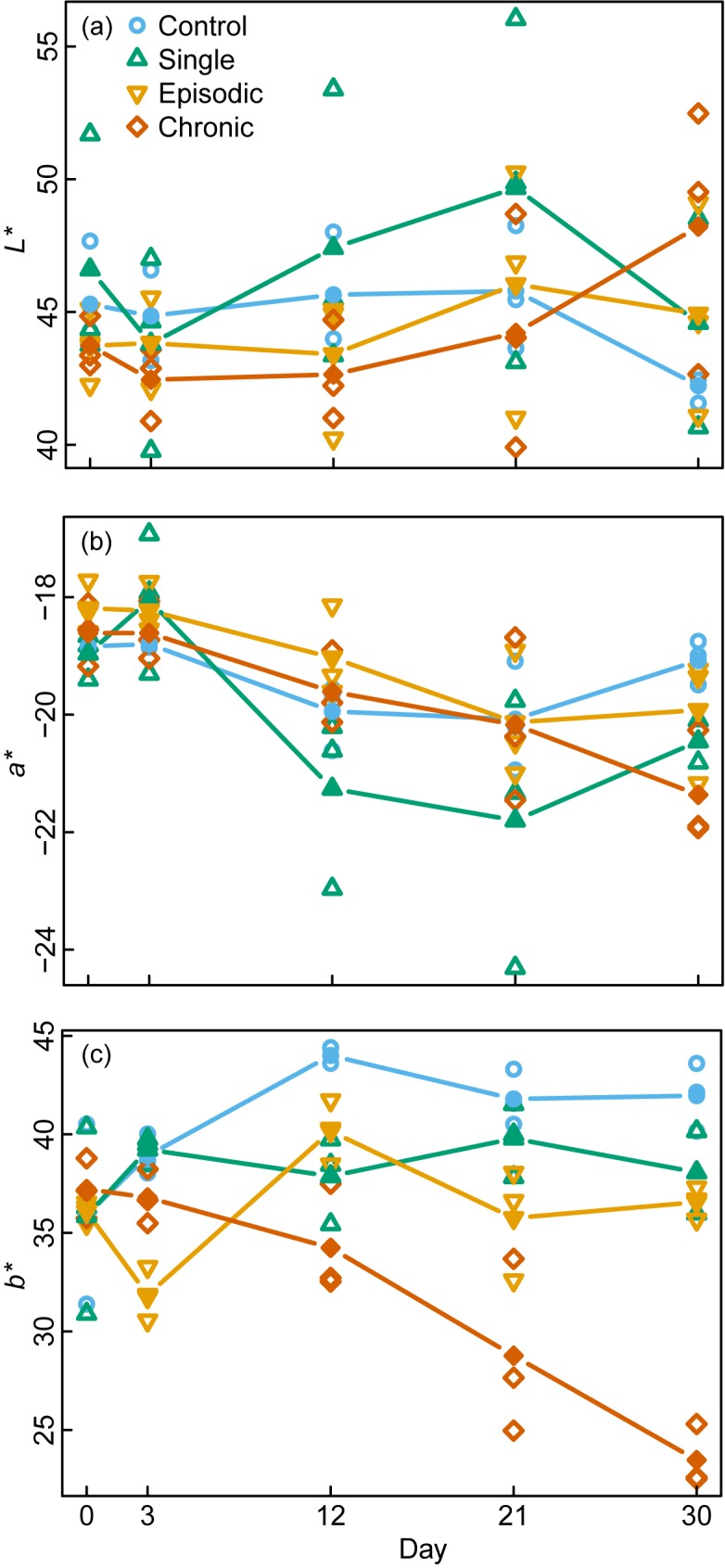
Repeated measurement of color values on *A*. *gibbosa* in response to different thermal-stress treatments. The CIE color space values (a) L* = whiteness, (b) a* = green (-) to magenta (+), (c) b* = blue (-) to yellow (+) at time zero and subsequently after each episodic thermal stress event. The different treatments are depicted according to: control / no stress (blue circles), single stress event (green triangles), episodic stress events (yellow inverse triangles) and chronic stress (red diamonds). Filled symbols connected by lines represent the running means of each treatment (n = 3) on the respective sampling time (day).

**Fig 4:**
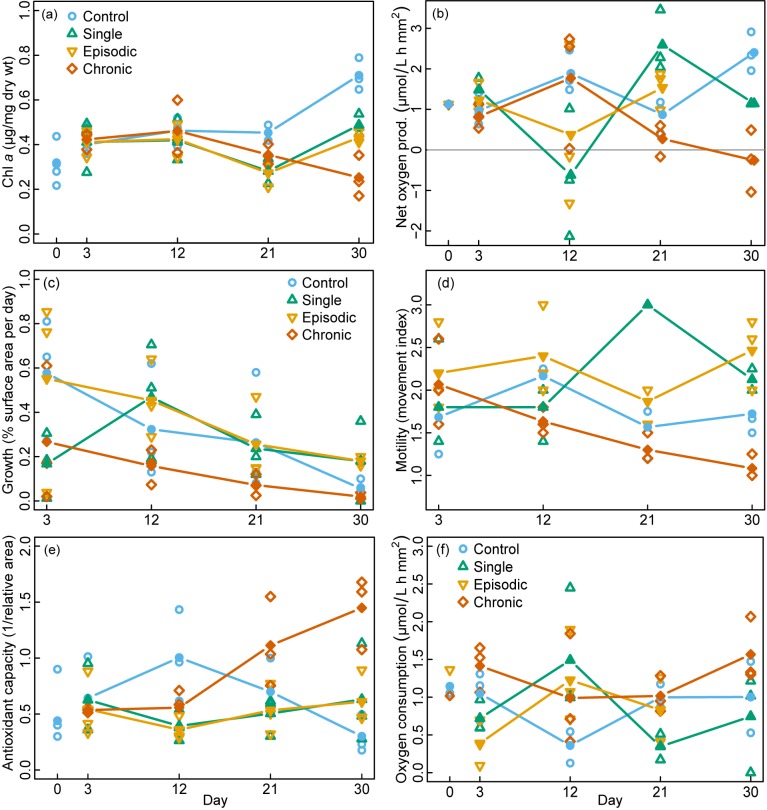
Repeated measurement of physiological variables on *A*. *gibbosa* in response to different thermal-stress treatments. (a) Chl *a* concentration (μg per mg dry wt), (b) net photosynthesis (expressed as oxygen-production rate per surface area), (c) growth rates (as a percentage of increase in surface area per day since the previous sampling time), (d) motility index indicating the amount of movement within the experimental vials, (e) total antioxidant capacity against peroxyl radicals (ACAP, expressed as the inverse of the relative area of fluorescence produced by reactive oxygen species), (f) respiration (expressed as oxygen-consumption rate per surface area) at time zero and subsequently after each episodic thermal-stress event. The different treatments are depicted according to: control / no stress (blue circles), single stress event (green triangles), episodic stress events (yellow inverse triangles) and chronic stress (red diamonds). Filled symbols connected by lines represent the running means of each treatment (n = 3) on the respective sampling time (day).

With respect to all photosymbiont-specific variables, the population exposed to chronic thermal stress showed the strongest response, including the highest frequency of bleaching and color changes, as well as the lowest Chl *a* concentration and oxygen-production rate at the end of the experiment (Tables 1 and 2, S1 Table, Figs 3 and 4B). The strength of the reaction increased with time. In contrast, the control treatment showed the least bleaching response, while oxygen production (net photosynthesis) increased, which is consistent with an increase in Chl *a* and colors over time (Table 1, Figs 3 and 4B, S2 Fig). The responses in treatments with single and episodic stress were remarkably similar with respect to all photosymbiont-specific parameters and remained stable through the duration of the experiment (Table 2, Figs 3 and 4B).

**Table 1:** Bleaching frequency of *A*. *gibbosa* exposed to different thermal-stress treatments.

Treatment	3 days	12 days	21 days	30 days
Control	0.07 ± 0.14	0.07 ± 0.14	0.13 ± 0.20	0.11 ± 0.18
Single	0.00 ± 0.00	0.35 ± 0.28	0.28 ± 0.26	0.33 ± 0.33
Episodic	0.07 ± 0.14	0.20 ± 0.23	0.27 ± 0.26	0.36 ± 0.28
Chronic	0.07 ± 0.14	0.22 ± 0.24	0.58 ± 0.28	0.93 ± 0.14

Average frequency and SE (n = 3) of the proportion of partial bleaching (mottling) of surviving specimens (see mortality in Table 3) out of 5 pooled individuals in response to exposure to the different treatments: control / no stress, a single stress event, episodic stress events or chronic stress, over different time periods.

**Table 2:** Repeated measures ANOVA of *A*. *gibbosa* exposed to different thermal-stress treatments.

Variable	Factor	df	F-ratio	*P*-value
L*	Treatment	3,8	0.48	0.707
	Time	4,32	3.82	**0.023**
	Treatment × Time	12,32	2.57	**0.031**
a*	Treatment	3,8	1.04	0.424
	Time	4,32	34.2	**< 0.001**
	Treatment × Time	12,32	4.88	**0.001**
b*	Treatment	3,8	56.6	**< 0.001**
	Time	4,32	8.71	**< 0.001**
	Treatment × Time	12,32	10.5	**< 0.001**
Chl *a*	Treatment	3,8	47.2	**< 0.001**
	Time	3,24	4.69	**0.010**
	Treatment × Time	9,24	4.20	**0.002**
Net photosynthesis^a^	Treatment	2,6	1.64	0.270
	Time	3,18	0.45	0.721
	Treatment × Time	6,18	10.2	**< 0.001**
Net photosynthesis^b^	Treatment	3,8	0.12	0.944
	Time	2,16	0.85	0.445
	Treatment × Time	6,16	4.83	**0.005**
Respiration^a^	Treatment	2,6	2.32	0.180
	Time	3,18	1.14	0.358
	Treatment × Time	6,18	3.09	**0.029**
Respiration^b^	Treatment	3,8	1.28	0.344
	Time	2,16	0.73	0.498
	Treatment × Time	6,16	3.39	**0.024**
ACAP	Treatment	3,8	3.04	0.093
	Time	3,24	1.55	0.229
	Treatment × Time	9,24	4.77	**0.001**
Growth	Treatment	3,8	5.05	**0.029**
	Time	3,24	3.92	**0.021**
	Treatment × Time	9,24	0.71	0.691
Motility	Treatment	3,8	6.85	**0.013**
	Time	3,24	0.12	0.945
	Treatment × Time	9,24	5.49	**< 0.001**

Results for the variables: motility, growth, CIE L*a*b* color space values, respiration, net photosynthesis, Chl *a*, and antioxidant capacity against peroxy radicals (ACAP) and different time periods. *P*-values <0.05 are printed in bold. The results of Tukey’s HSD post hoc test for all variables that showed significant interactions between Treatment × Time that further identifies individual differences between treatments at each sampling day are found in S1 Table.

^a^ ANOVA was performed only for control, single- and chronic-stress treatments on all sampling times.

^b^ ANOVA was performed for all treatments but only on sampling times 3, 12 and 21.

The reduced performance of the photosymbionts consistently seen in the chronic exposure treatment was mirrored by all holobiont variables. The chronic treatment showed the lowest growth rates and motility as well as the highest ACAP (Table 2, S1 Table, Fig 4C–4E). These variables displayed a clear temporal trend. Oxygen consumption (respiration) showed no significant differences among the treatments and no trend (Fig 4F). As in the photosymbiont-specific variables, the holobiont response appeared to be similar for the single- and episodic-stress treatments (Table 2, Fig 4C–4E). In most variables, the response of these treatments was comparable to the control, except for motility, which was significantly higher in the single- and episodic-stress treatments, and the ACAP, which showed different temporal trends. The single-stress treatment and the control also displayed similar mortality trends (Table 3, S1 Fig). Because empty shells were associated with treatments where photosymbiont and holobiont variables indicated optimum growth, and because the experiment was conducted at the time of year when these populations normally reproduce [33], the presence of empty shells in this study is interpreted as being caused by reproduction. Because the interpretation of mortality as reproduction could not be assured and quantified, it has to be treated with caution. Since most standard errors are high, trends in the running mean should not be over interpreted.

**Table 3:** Mortality of *A*. *gibbosa* exposed to different thermal-stress treatments.

Treatment	3 days	12 days	21 days	30 days
Control	0.00 ± 0.00	0.13 ± 0.20	0.13 ± 0.20	0.33 ± 0.27
Single	0.00 ± 0.00	0.07 ± 0.14	0.27 ± 0.26	0.53 ± 0.29
Episodic	0.00 ± 0.00	0.00 ± 0.00	0.00 ± 0.00	0.00 ± 0.00
Chronic	0.00 ± 0.00	0.00 ± 0.00	0.00 ± 0.00	0.00 ± 0.00

Average frequency and SE (n = 3) of the proportion of mortality (empty shells) out of 5 pooled individuals in response to exposure to the different treatments: control / no stress, a single stress event, episodic stress events or chronic stress, over different time periods.

**Fig 5:**
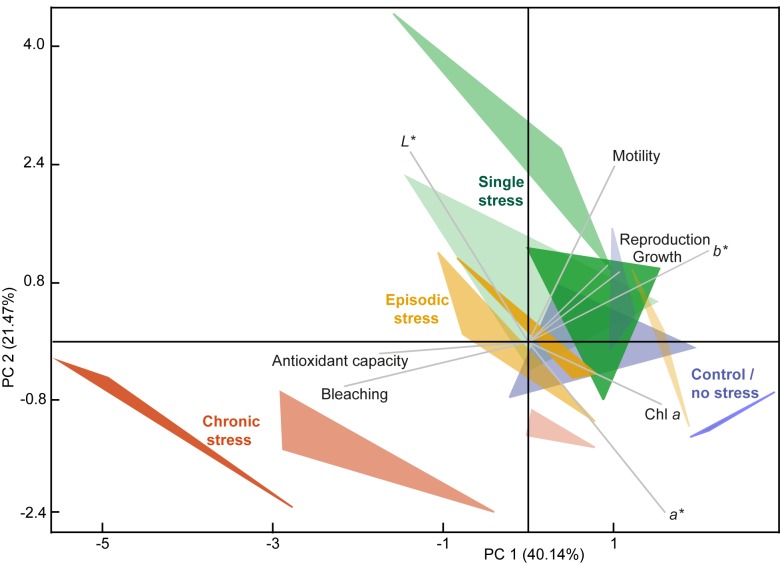
Principal component analysis (PCA) biplot visualizing experimental stress response patterns. The corners of the triangles represent the three replicates per thermal treatment: control / no stress (blue), single stress event (green), episodic stress events (yellow) and chronic stress (red); and the color intensity represents the temporal variable from day 12 (transparent), to day 21 and the final sampling time at day 30 (solid). The influence of partial bleaching frequency, CIE L*a*b* color space values (L* = whiteness, a* = green to magenta, b* = blue to yellow), Chl *a* concentration, antioxidant capacity against peroxyl radicals (ACAP), growth rate, motility and mortality seen as empty shells (most likely indicating reproduction) are directionally indicated.

The overall pattern of stress response among the treatments through time is visualized by a principal component analysis (PCA, Fig 5). The PCA portrays nine variables from day 12 to day 30 and reveals high collinearity in the response variables reflected in the fact that the first two principal components account for >60% of total variance. The biplot of these two principal components highlights the fundamental differences in directional response of the chronic treatment, which shows a stronger response with time, associated with ACAP and variables describing bleaching. In contrast, the single treatment and the episodic treatment appear to behave in a similar way. They show no unidirectional trend in their response and deviate from the control only due to higher motility and no significant increase in Chl *a* through time. The biplot also illustrates the consistency among the replicates of the treatments, which seems to show decreasing variance through time. The larger fluctuations in the response through time observed in the single-stress treatment have to be seen in the light of the highest frequency of empty shells (mortality most likely due to reproduction) in this treatment, implying that a large part of this variability could be due to high motility and color changes prior to reproduction.

## Discussion

The results demonstrate that the physiological effects of single and episodic stress events on photosymbiotic calcifiers are markedly different compared to chronic stress. Single and episodic thermal peaks did not impair the function of *A*. *gibbosa*, while chronic stress damaged the algal photosymbionts, induced an antioxidant defense response, and compromised the overall holobiont health and activity. The divergence in physiological responses between the chronic and episodic thermal stress seems to have developed between day 3 and day 12 of the experiment (Figs 3 and 4). This divergence emphasizes not only the temporal tipping point and damage associated with chronic stress but also the importance of respite phases during thermal stress. The temperature conditions in this experiment emulate water temperature variability and duration (hours to days) shifts >5°C recorded in tropical reefs [18, 29] and therefore present real-life scenarios of temperature stress.

### Control treatment

Since the photosymbionts in the control treatment flourished, the host grew, reproduction likely occurred (seen as mortality), and ACAP values did not rise above natural population averages, we can use the experimental conditions and observed response patterns to predict how field populations respond. For instance, Chl *a* concentration increased over time, indicated by lower L* and increasing b* values, as well as greater net oxygen-production rates by the end of the experiment (S2 Fig). This response can be explained by natural increase in numbers of photosymbionts during the ontogeny of the foraminifera and may also reflect an increase symbiont density in response to low light levels in culture conditions [19, 49] or a possible feeding-related rise in the availability of fixed nitrogen, which could increase the amount of nutrients supplied from the host to the symbionts. The former process of photo-acclimation is known from corals, which can increase the amount of chloroplasts in their photosymbionts to meet their energy demands despite low-light conditions in their environment [50] and might act similarly in LBF by increasing the amount of symbionts or their chloroplasts.

The ACAP values of the control population are comparable to values reported for a population of *A*. *lobifera* in the Great Barrier Reef, which shows elevated resilience towards temperature and nutrient stress, probably due to preconditioning based on environmental fluctuations [23]. Specifically, both the absolute ACAP values and the temporal trend in the control resemble those measured by Prazeres et al. [23], indicating that the population of *A*. *gibbosa* in our study is possibly acclimatized or adapted to comparable conditions.

### Chronic thermal stress

Chronic thermal stress induced gradual bleaching, which is reflected by reduced photopigment concentrations and ultimately decreasing photophysiological performance. This observation is in line with previous studies on LBF [14, 16, 17, 19, 23]. Although oxygen-production rates were negative after 30 days of chronic stress exposure, holobiont respiration rates indicated that the remaining photosymbionts were still photosynthetically active (S2 Fig). Those specimens that exhibited intense bleaching showed accumulation of brown material at the periphery of the shell and close to the aperture (S1D Fig) resulting from the deterioration of chloroplasts, typically followed by degradation or expulsion of the photosymbiont residues [17, 19]. Despite survival of some photosymbionts, their decreased concentration and activity likely impaired the fitness of the holobiont, by reduced translocation of metabolites causing lower growth rates [15–17, 21, 22], reduced motility [17] and probably also less reproductive activity [24] (here seen as mortality). Although growth rates across all treatments gradually slowed this is most likely due to the same natural aging trends known for benthic foraminifera [51]. It is remarkable that that the chronic-stress treatment seems to have reduced growth by ~50% in comparison to the other treatments after the first measurements in the treatment. This early reduction in growth indicates that the primary response to chronic thermal stress is likely due to the holobionts using their energy to maintain homeostasis. Respiration rates could indicate that bacteria, which were feeding on the remains of dead foraminifers were respiring very actively. Alternatively, the respiration rates could indicate that *A*. *gibbosa* specimens from the chronic-stress treatment were still alive at the end of the experiment, although they did not reproduce, ceased to move, and did not grow after 21 days of chronic exposure.

Together with previous studies, our results support the hypothesis that foraminiferal hosts are more resistant to thermal stress than their endosymbionts [19, 40]. Cytological analyses revealed that prolonged temperature stress under low light conditions (6–8 μmol photons m^-2^ s^-1^) induced significant declines in photosymbiont densities and lipid bodies, while some host endoplasm remained intact [19]. In our experiment, similar exposure temperature and duration (32°C for one month) led to bleaching but was sub-lethal to the host, which reconfirms that LBF can survive bleaching, however with the overall reallocation of metabolic activity.

The lack of mortality in the chronic-stress treatment in our experiment seems to be at odds with other long-term chronic exposure studies, which showed increased mortality at elevated temperatures [16, 22, 23]. This could be related to variations between LBF and photosymbiont species, or durations and intensities of stress exposure in the different studies. However, the functionality of the holobiont at the end of our experiment appears to have been so severely impaired that more profound effects will likely ensue if stress continues or other interacting pressures occur [22]. Hallock et al. [33] reported a variety of issues associated with bleaching in *A*. *gibbosa*, including reproductive failure, epibiont infestations and calcification anomalies.

Here, we show for the first time that ACAP in *A*. *gibbosa* is greatly enhanced by chronic thermal stress (Figs 4E and 5). The only other study to measure ACAP in LBF in response to thermal stress showed that after 30 days at 29°C, the ACAP of *A*. *lobifera* had not increased significantly [23]. The lack of ACAP response from *A*. *lobifera* compared to *A*. *gibbosa* from our study may have resulted from the 3°C higher exposure temperature in our experiment, species-specific temperature tolerances, different local adaptations or symbiont communities. The function of elevated ACAP is associated with defense mechanisms against amplified oxygen radicals produced by photosynthesis under higher temperature, as seen amongst cnidarians [52]. Although the density of symbionts, which are expected to produce radical oxygen species, decreased over time (e.g. Fig 4A) the ACAP increased continuously. This implies that either the remaining but more and more damaged symbionts were still producing sufficient oxygen radicals for the host’s defense system to require higher ACAP to compensate for these, or that the antioxidant capacity was responding to the oxidative stress with a time lag. Since we did not measure gene or protein expression as in other studies [20, 27], but on the level of enzyme and non enzymatic low-molecular-weight scavenger (e.g. glutathione, ascorbic acid, uric acid, vitamin E and carotenoids) capacity [42, 52], these might be produced more slowly and, more importantly, might remain functional over considerably longer time periods. Overall, our chronic stress scenario suggests a reallocation of host energy towards defense and repair mechanisms, thereby reducing calcification, motility and reproductive activity but preventing mortality.

### Single and episodic stress events

The *A*. *gibbosa* coped well with fluctuating temperatures simulated by single and episodic thermal stress events. Most photosymbiont and holobiont response variables did not change significantly over the term of the experiment. This seems contradictory to former studies that analyzed the responses of LBF to short-term thermal stress, which found lower Chl *a* concentrations, reduced photosynthetic efficiency [16, 17], and lower quantities of RuBisCO [20] after hours to days of exposure. These studies, however, focused on the immediate response to stress, while our results represent their physiological response after they were released from the thermal stress. It is therefore possible that *A*. *gibbosa* and most of the photosymbiont variables (e.g., Chl *a* and color values) had already recovered within 24 hours after the peak thermal stress, demonstrating the capability of this species to quickly recover from short-term stress. Similarly, oxygen-consumption rates required only a few hours to recover from extreme temperatures, in contrast to photosynthesis rates that needed several days to recover [40].

After the single stress event, net photosynthesis varied strongly over time. Because these variations were ongoing throughout the experiment, we interpret them as most likely related to the presumably high incidence of reproduction in this treatment (data lacking). Reproductive activity even exceeded the control specimens and represented the only variable in which single stress and episodic stress responses differed. Because half of the shells in the single-stress treatment were empty by the end of the experiment, the single thermal peak followed by stable conditions might have stimulated reproduction. In contrast, episodic stress appeared to suppress or delay reproduction in the same way as in the chronic-stress treatment. Correspondingly, suppression of asexual reproduction in adults and failure to normally calcify were reported from *A*. *gibbosa* specimens collected during summer, which also exhibited photosymbiont deterioration [33]. Previous studies [24, 51] related reduced reproduction and fecundity to low light intensities. Since in our study the light level was the same in all aquaria and reproduction presumably occurred in other treatments, this does not seem to be the driving factor here. In the case that recurring stress induces malfunction or impairment of reproductive activities, this would imply important long-term consequences for foraminiferal life cycles, population densities and community structures with severe impacts on carbonate budgets and overall health of coral-reef environments [10, 24, 33, 41] and should be addressed in future studies.

### Analogy to other coral reef calcifiers

While there are no comparable studies on the effects of fluctuating temperatures on LBF, other photosymbiotic reef organisms have been subjected to temperature variations and showed that even short temperature reductions can reduce immediate thermal damage within coral reefs. Such examples include large-amplitude internal waves, which cause pH and temperature to drop within minutes, allowing short-term relief, and have been shown to reduce the physiological effect of heat stress on corals [30]. Daily temperature fluctuations can be beneficial to the photosynthetic efficiency of coral larvae [26], but led to strong declines in photosymbiont densities, while maintaining or even increasing calcification in studies on adult coral colonies [53]. Corals that are exposed to extreme natural temperature fluctuations during spring-tide upwelling events increase most physiological and molecular parameters, suggesting that the holobiont may acclimate to fluctuating temperatures by the symbionts capacity to increase photosynthesis and carbon fixation [27]. These results and our study support the hypothesis that temperature fluctuations, in contrast to chronic thermal stress, have substantially different effects on photosymbiotic reef calcifiers. The impact of thermal stress appears to not only depend on exposure level and duration, but also on whether the stress is constant or discontinuous because intermittent stress provides respite periods permitting repair mechanisms to alleviate or entirely prevent the detrimental effects of thermal stress. Interactive effects of multiple contemporaneous or consecutive stressors could produce different outcomes and should be targeted by future research.

Besides the immediate effects of temperature variations, thermal history is an important factor among photosymbiotic reef organisms, because local acclimatization or adaptation to thermal stress may enhance thermal resistance through higher phenotypic and metabolic plasticity. This is evident by elevated thermal tolerance in corals from habitats where they naturally experience temperature fluctuations, such as large-amplitude internal waves [30] or lagoon pools [7]. Furthermore, coral colonies that were experimentally pre-stressed before exposure to severe prolonged thermal stress revealed more effective photoprotective mechanisms [8]. Similar to coral studies, *A*. *lobifera* populations from stable offshore environments are more sensitive to stress than those from inshore habitats that experience stronger fluctuating conditions [23]. Comparably, our results indicate that local conditions increased the tolerance of *A*. *gibbosa* to environmental changes, considering long-term subsurface temperature variability in the Florida Keys [29]. Specifically at the sampling location, Tennessee Reef situated in the Middle Keys, reefs were historically exposed to intermediate levels of sea-surface temperature variability [54]. These intermediate thermal fluctuations seem to be beneficial to biodiversity, survival, and recovery of the local stony-coral assemblages [28]. It is therefore highly probable that the population of *A*. *gibbosa* sampled for our experiment is adapted or acclimatized to thermal variability such that single- and episodic-stress treatments did not exceed its tolerance range. Indeed, time-series studies of *A*. *gibbosa* populations from the Florida Keys through the 1990s revealed that bleaching followed the solar cycle of irradiance, such that peak bleaching consistently occurred well before the late summer temperature maximum and the populations were typically already showing recovery when temperature peaked [4, 33].

No acclimatization to repeated stress events occurred in our study, but the LBF under chronic stress arrived close to the thermal tipping point. In some corals, elevated thermal tolerance can be independent of local variation in ocean temperature, such that their acclimatization capacity to future warming is limited [32]. Whether *A*. *gibbosa* is generally characterized by high thermal tolerance or if the high physiological plasticity found in this study is specific to the local population assessed, which would suggest a high acclimatization capacity, has to be targeted in future research. This raises the discussion on whether the resilience of these foraminifers is a product of short-term acclimatization due to recent thermal history, or if long-term adaptation has increased the tolerance of these photosymbiotic calcifiers. Such questions could be disentangled with the use of ‘omics’ approaches, which can determine the influence of environmental stressors on the gene or protein level and therefore reveal meaningful insights into underlying molecular processes governing acclimatization/adaptation pathways. Furthermore, research on the flexibility and physiological plasticity of the photosymbiont community would further improve our understanding of LBF adaptive potential.

## Conclusions

Our laboratory experiment represents the first study focusing on the physiological responses of LBF to temperature fluctuations. Although some physiological responses showed high variability, this study illustrates how thermal variation has different effects on the foraminifera and their photosymbionts compared to chronic exposure despite the same peak temperature. We also showed how reoccurring stress did not induce acclimatization, likely because *A*. *gibbosa* populations from the Florida Keys are already adapted to the applied pattern and amount of temperature variability. This study, together with coral research, conveys how temperature fluctuations affect reef ecosystems differently than chronic exposure, provided that the intensity and duration of transient thermal stress events do not exceed naturally occurring extremes [28, 54]. This study further demonstrates that experimental studies and projections of global change effects on reef calcifiers must consider temporal fluctuations in stress exposure. In a warming ocean, fluctuations in stress level can be an important factor to facilitate recovery from chronic heat stress [30], which either allow for short-term acclimatization [27], or induce physiological acclimation [8] by enhancing metabolic efficiency [26]. The energetic costs of acclimatization through high physiological plasticity [18], such as possible suppression of reproduction, are important aspects that need to be addressed in future research. Overall, marine habitats with fluctuating temperature regimes may bear highly resilient reef calcifiers with a high potential to seed or serve as potential reef refugia [28–30], and therefore need to be primary focal points of coral reef research to guide global conservation efforts.

## Supporting information

S1 Table Results of Tukey’s HSD post hoc test for treatment × time interactions. All variables that showed significant interactions between Treatment × Time in the ANOVA (*P*-value < 0.05 in Table 2) were followed by Tukey's HSD test procedure to further identify individual differences between treatments (1 = control / no stress, 2 = single stress event, 3 = episodic stress events, 4 = chronic stress) at each sampling day (time). For respiration and net photosynthesis the ANOVA was performed (a) only for treatments 1, 2 and 4 on all sampling days or (b) only on sampling days 3, 12 and 21 for all treatments.
(PDF)

S1 Fig Representative photos of *Amphistegina gibbosa* after exposure to different thermal-stress treatments for 30 days. The images illustrate changes in holobiont color and appearance of empty shells, representing one of the three replicates in each of the treatments: (a) control / no stress, (b) single stress event, (c) episodic stress and (d) chronic stress. Individuals in (a) and (b) that turned entirely white (empty tests) died or underwent reproduction, some specimens in (b) and (c) showed mottling / partial bleaching and severely impacted foraminifera in (d) bleached strongly, but at the same time showed accumulation of dark materials at the shell periphery. White scale bars represent 1 mm length.
(PDF)

S2 Fig Gross photosynthesis rates from *A*. *gibbosa* in response to different thermal-stress treatments. The gross photosynthesis is express as oxygen-production rate per surface area at time zero and subsequently after each episodic thermal-stress event in the treatments: control / no stress (blue circles), single stress event (green triangles), episodic stress events (yellow inverse triangles) and chronic stress (red diamonds). Filled symbols connected by lines represent the running means of each treatment (n = 3) on the respective sampling time (day).
(PDF)
