# Supplementary material for: Reef calcifiers are adapted to episodic heat stress but vulnerable to sustained warming
Source: PLoS One. 2017 Jul 6;12(7):e0179753. doi: 10.1371/journal.pone.0179753 (PMC5500281; doi:10.1371/journal.pone.0179753)
Supplement: S1 Table — All variables that showed significant interactions between Treatment × Time in the ANOVA (P-value < 0.05 in Table 2) were followed by Tukey's HSD test procedure to further identify individual differences between treatments (1 = control / no stress, 2 = single stress event, 3 = episodic stress events, 4 = chronic stress) at each sampling day (time). For respiration and net photosynthesis the ANOVA was performed (a) only for treatments 1, 2 and 4 on all sampling days or (b) only on sampling days 3, 12 and 21 for all treatments. (PDF) [file pone.0179753.s001.pdf]

S1 Table. Results of Tukey's HSD post hoc test for Treatment × Time interactions.

Variable	Time (day)	Treatment	Estimate	SE	t-ratio	*P*-value
*b**	3	1 - 3	7.063	1.643	4.30	0.001
		2 - 3	7.379	1.643	4.49	0.001
		3 - 4	-4.944	1.643	-3.01	0.025
	12	1 - 2	6.124	1.643	3.73	0.004
		1 - 4	9.757	1.643	5.94	< 0.001
		3 - 4	5.914	1.643	3.60	0.006
	21	1 - 3	6.044	1.643	3.68	0.005
		1 - 4	13.028	1.643	7.93	< 0.001
		2 - 4	11.019	1.643	6.71	< 0.001
		3 - 4	6.984	1.643	4.25	0.001
	30	1 - 3	5.406	1.643	3.29	0.012
		1 - 4	18.490	1.643	11.25	< 0.001
		2 - 4	14.520	1.643	8.84	< 0.001
		3 - 4	13.084	1.643	7.96	< 0.001
Chl *a*	21	1 - 2	0.208	0.060	3.45	0.010
		1 - 3	0.217	0.060	3.60	0.007
	30	1 - 2	0.223	0.060	3.70	0.005
		1 - 3	0.278	0.060	4.62	0.001
		1 - 4	0.457	0.060	7.59	0.000
		2 - 4	0.235	0.060	3.90	0.003
		3 - 4	0.179	0.060	2.98	0.030
Net photosynthesis[a]	12	1 - 2	2.508	0.701	3.58	0.006
		2 - 4	-2.393	0.701	-3.41	0.009
	21	2 - 4	2.321	0.701	3.31	0.011
	30	1 - 4	2.661	0.701	3.79	0.004
		2 - 4	2.173	0.701	3.10	0.017
Net photosynthesis[b]	12	1 - 2	2.508	0.771	3.25	0.017
		2 - 4	-2.393	0.771	-3.11	0.024
	21	2 - 4	2.321	0.771	3.01	0.029
Respiration[a]	12	1 - 2	1.131	0.364	3.11	0.013
Respiration[b]	3	3 - 4	1.025	0.358	2.87	0.040
	12	1 - 2	1.131	0.358	3.16	0.021
	21	1 - 2	0.208	0.060	3.45	0.010
ACAP	12	1 - 3	0.645	0.226	2.85	0.039
	30	1 - 4	-1.147	0.226	-5.08	0.000
		2 - 4	-0.818	0.226	-3.62	0.006
		3 - 4	-0.838	0.226	-3.71	0.005
Motility	21	1 - 2	-1.433	0.311	-4.61	< 0.001
		2 - 3	1.133	0.311	3.64	0.005
		2 - 4	1.700	0.311	5.47	< 0.001
	30	2 - 4	1.333	0.311	4.29	0.001
		3 - 4	1.383	0.311	4.45	0.001
