# Supplementary material for: Reef calcifiers are adapted to episodic heat stress but vulnerable to sustained warming
Source: PLoS One. 2017 Jul 6;12(7):e0179753. doi: 10.1371/journal.pone.0179753 (PMC5500281; doi:10.1371/journal.pone.0179753)
Supplement: S1 Fig — The images illustrate changes in holobiont color and appearance of empty shells, representing one of the three replicates in each of the treatments: (a) control / no stress, (b) single stress event, (c) episodic stress and (d) chronic stress. Individuals in (a) and (b) that turned entirely white (empty tests) died or underwent reproduction, some specimens in (b) and (c) showed mottling / partial bleaching and severely impacted foraminifera in (d) bleached strongly, but at the same time showed accumulation of dark materials at the shell periphery. White scale bars represent 1 mm length. (PDF) [file pone.0179753.s002.pdf]

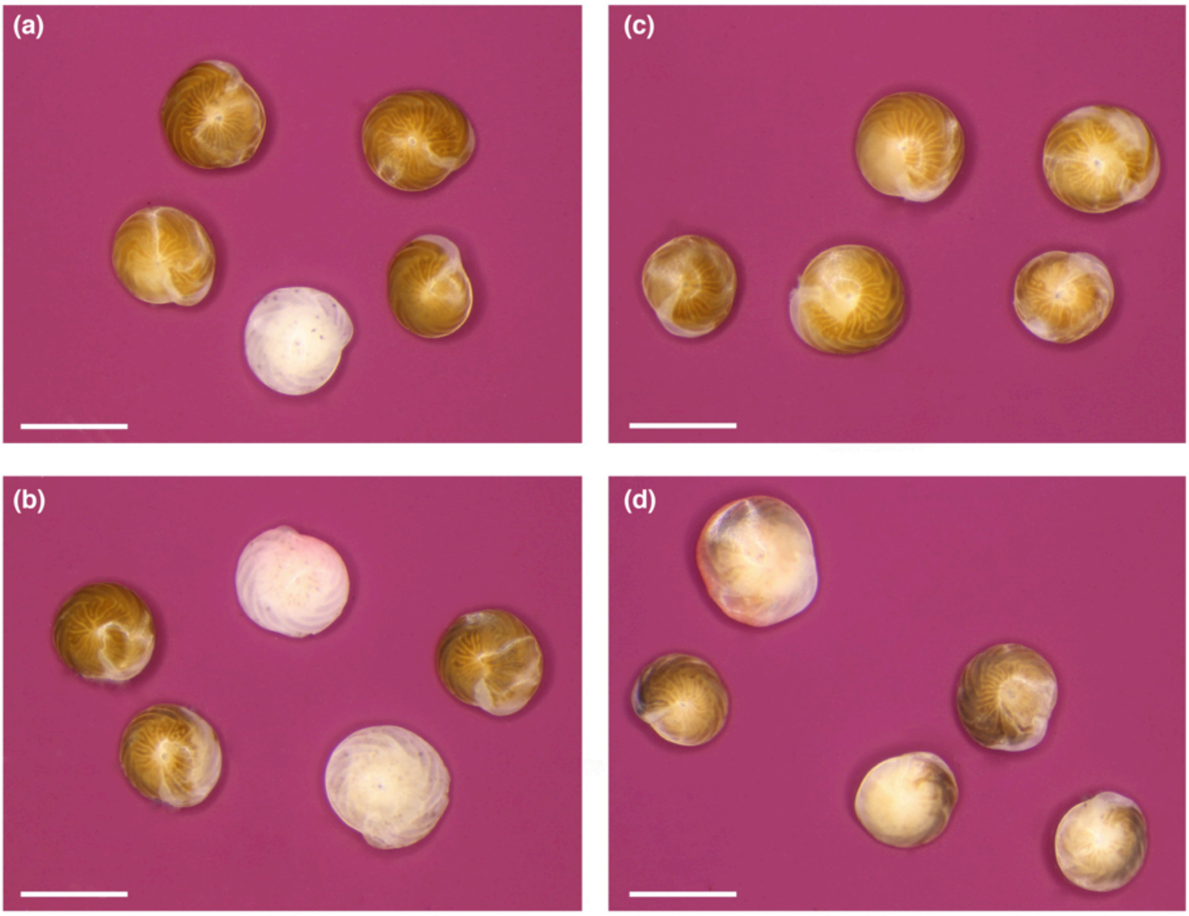

S1 Fig. Representative photos of *Amphistegina gibbosa* after exposure to different thermal-stress treatments for 30 days. The images illustrate changes in holobiont color and appearance of empty shells, representing one of the three replicates in each of the treatments: (a) control / no stress, (b) single stress event, (c) episodic stress and (d) chronic stress. Individuals in (a) and (b) that turned entirely white (empty tests) died or underwent reproduction, some specimens in (b) and (c) showed mottling / partial bleaching and severely impacted foraminifera in (d) bleached strongly, but at the same time showed accumulation of dark materials at the shell periphery. White scale bars represent 1 mm length.
