# Supplementary material for: Reef calcifiers are adapted to episodic heat stress but vulnerable to sustained warming
Source: PLoS One. 2017 Jul 6;12(7):e0179753. doi: 10.1371/journal.pone.0179753 (PMC5500281; doi:10.1371/journal.pone.0179753)
Supplement: S2 Fig — The gross photosynthesis is express as oxygen-production rate per surface area at time zero and subsequently after each episodic thermal-stress event in the treatments: control / no stress (blue circles), single stress event (green triangles), episodic stress events (yellow inverse triangles) and chronic stress (red diamonds). Filled symbols connected by lines represent the running means of each treatment (n = 3) on the respective sampling time (day). (PDF) [file pone.0179753.s003.pdf]

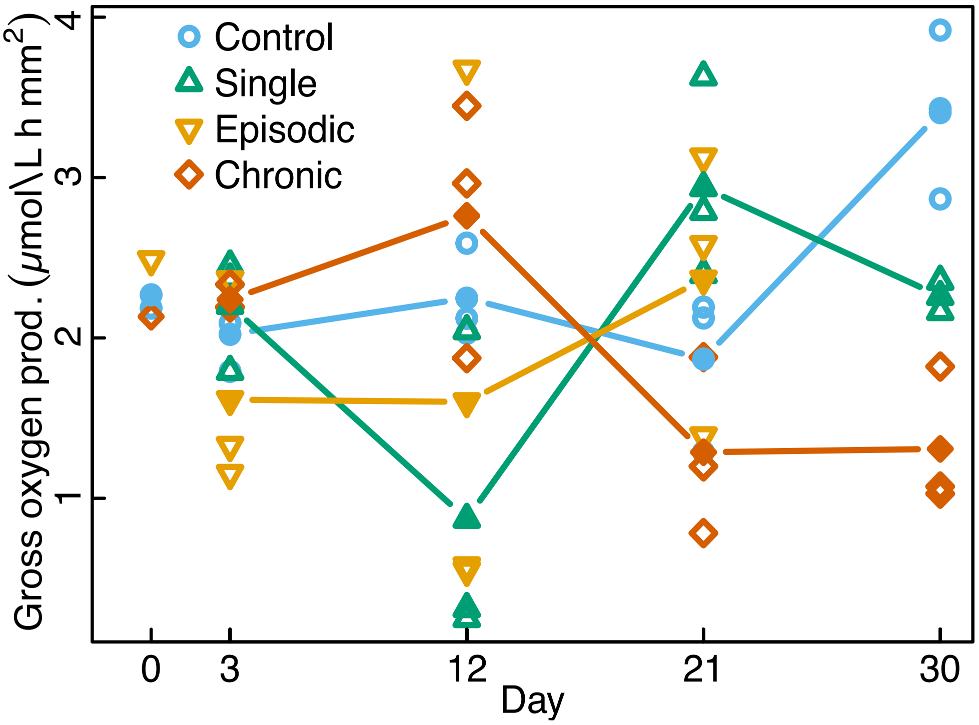

S2 Fig. Gross photosynthesis rates of *A. gibbosa* in response to different thermal-stress treatments. The gross photosynthesis is express as oxygen production per surface area at time zero and subsequently after each episodic thermal-stress event in the treatments: control / no stress (blue circles), single stress event (green triangles), episodic stress events (yellow inverse triangles) and chronic stress (red diamonds). Filled symbols connected by lines represent the running means of each treatment (n= 3) on the respective sampling time (day).

! "
